# Practical Pharmacokinetic–Pharmacodynamic Models in Oncology

**DOI:** 10.3390/pharmaceutics17111452

**Published:** 2025-11-11

**Authors:** Su Guan, Mei-Juan Tu, Ai-Ming Yu

**Affiliations:** Department of Biochemistry and Molecular Medicine, School of Medicine, University of California at Davis, Sacramento, CA 95817, USA; suguan@health.ucdavis.edu (S.G.); mjttu@health.ucdavis.edu (M.-J.T.)

**Keywords:** pharmacokinetic, pharmacodynamic, model, oncology, drug combination

## Abstract

Integrated pharmacokinetic (PK) and pharmacodynamic (PD) models are essential for the understanding of quantitative relationship between drug exposure and response towards the identification of optimal dosing regimens in drug development and clinical therapy. This article summarizes the common PK–PD models being established in oncology, with a focus on combination therapies. Among them, the PK models include those used for practical non-compartmental and compartmental analyses, as well as those for physiologically based modeling that describe and predict exposure to various chemotherapy, targeted therapy, and immunotherapy drugs. Built on proper natural disease progression models, such as the empirical logistic growth curve, the Gompertzian growth model, and their modifications, the integrated PK–PD models recapitulate and predict antitumor drug efficacy, in which the PD models include practical indirect response model and various tumor growth inhibition models, as driven by the mechanistic actions of the drugs administered. Since anticancer drugs are usually co-administered, PK–PD modeling has been extended from monotherapy to combination therapy. However, relying on a single interaction factor or parameter to capitulate complex drug interactions, predict outcomes of different combinations, and determine possible synergism is problematic. Considering the apparent contributions from individual drugs following mutual interactions, a new PK–PD model has been developed for combination therapy, which may be integrated with proper algorism (e.g., the Combination Index method) to critically define combination effects, synergism, additivity, or antagonism. As drug combinations become more complex and individual drug actions are variable, these models should be optimized further to advance the understanding of PK–PD relationships and facilitate the development of improved therapies.

## 1. Introduction

Anticancer drugs have historically shown moderate success rates, with only approximately half of the candidates progressing from Phase II into more resource-intensive Phase III clinical trials [1,2]. During Phase III trials, these drug candidates encounter another 50% chance of failure, underscoring the critical need for more efficient approaches in anticancer drug development [3,4,5]. Integrated pharmacokinetics (PK) and pharmacodynamics (PD) modeling has been proven as one of many powerful approaches in anticancer drug development by establishing quantitative drug exposure–response relationship towards the identification of proper dosing regimens to achieve optimal therapeutic outcomes [6,7,8,9,10,11].

The PK model describes the behavior of drug absorption, distribution, metabolism, and excretion, and PD focuses on its pharmacological effects, from the molecular and cellular levels to the systems [10,11]. By integrating the two domains, a PK–PD model enables the characterization of quantitative relationship between drug exposure and mechanistic response (therapeutic or adverse effects), facilitating the identification of drug dosing regimen towards optimal efficacy and minimal toxicity [7,11,12]. In oncology, where the therapeutic window is often narrow, a precise modeling of PK–PD relationship is essential to understand and balance the efficacy and safety [12,13,14,15,16].

Regulatory agencies such as the United States Food and Drug Administration (FDA) have recognized the utility of PK–PD modeling and simulation in enhancing clinical trial designs and decision-making processes. Indeed, Project Optimus, launched by the FDA, emphasizes innovative trial methodologies, including PK–PD modeling, to improve dose optimization in oncology drug development [17,18]. Computational model-based approaches integrate natural disease progression models, PK, and PD to optimize trial designs, enhance quantitative decision-making, and support therapeutic regimen development. These models enable quantitative characterization of drug exposure–response relationships while accounting for natural disease progressions and drug actions [13,19,20,21,22,23,24,25,26].

Combination therapy is very common in oncology, particularly for patients with advanced cancers [27,28,29]. The use of two or multiple drugs, either in the category of chemotherapy, targeted therapy or immunotherapy, aims to enhance treatment efficacy while mitigating adverse effects, whereas it introduces complexities in understanding the interactions of combined drugs as well as contributions of individual drugs to final outcomes. Characterizing the dynamics and potency of drug interactions in vitro remains challenging, and it is even harder for in vivo effects due to the interplay with disease progression [30,31,32]. A variety of PK–PD models have been developed to delineate the antitumor efficacy of combination therapies by introducing some factors to recapitulate interactions of co-administered drugs or their apparent contributions to combination therapy [22,33,34,35,36,37]. These models are instrumental in evaluating drug interactions and their impact on overall combination outcomes.

This review article is to summarize the current landscape of integrated PK–PD models in oncology, including those for mono- and combination therapies as well as their applications. Specifically, we highlight monotherapy PK–PD models as the foundation for characterizing exposure–response relationships of single agents, and combination PK–PD models as extensions that incorporate drug–drug interactions and interaction algorithms. By pointing out the interconnection, but also conceptual differences between drug interactions and combination synergism, the limitations of existing combination therapy PK–PD models are discussed, and caution is advised for the evaluation of potential combination synergism without using a valid algorithm. The discussions align with the values of PK–PD modeling to facilitate drug development and clinical therapy.

## 2. PK Models to Describe and Predict Drug Exposure

PK modeling is crucial for quantitative determination of drug level and duration within a target system, with the knowledge or assumption of dosing, input rates, and elimination properties. Several models have been established and widely used to characterize the PK profiles of various drugs, administered as individual agents or in combination [38,39]. Commonly used PK models can be categorized into three major classes, noncompartmental, compartmental, and physiological models, each offering distinct advantages, depending on the available data and research questions [13].

Non-compartmental analysis (NCA) is a straightforward approach where the PK parameters are directly calculated from the data collected during the study [13,40], without attempting to predict drug exposure under different circumstances. For instance, C_max_ (the maximum drug concentration) is simply the highest concentration observed in the collected data, and T_max_ (the time at which C_max_ occurs) is the associated time point. A key issue with NCA arises if the data around T_max_ is not sufficiently dense, as the true C_max_ may be missed. Additionally, if the dose is changed (e.g., halved), a fresh set of data would be required to answer questions about whether or how T_max_ might be changed. The main benefit of NCA is its simplicity and ease of implementation. It does not require solving complex differential equations, making it a quicker and more resource-efficient method [41,42,43]. NCA is often employed as an initial approach to assess apparent PK parameters like systemic clearance (CL) and volume of distribution (V_d_). It is also valuable for evaluating properties such as linearity and stationarity in drug behavior. The main drawbacks of NCA include the requirement for high data density to ensure accuracy and the fact that an NCA model is not predictive [44]. As such, it cannot predict how the behaviors of a drug would change in different scenarios. This makes NCA less useful in the later stages of drug development when more predictive and mechanistic models are often needed.

Different from a simple extraction of results from the data by NCA, compartmental analysis involves the creation of a theoretical model to recapitulate how a drug moves through the body and then fitting the observed data to this model. In this approach, the body is divided into hypothetical “compartments”, which represent areas where the drug can distribute and from which it can be eliminated [41,45]. Based on this view, scientists make certain assumptions and develop models based upon nonlinear regression analyses to describe the PK of the drugs. While a compartmental analysis can theoretically include any number of compartments, most PK data, including the entire concentration-time profiles are effectively modeled using one or two compartments [46,47]. From this fitted model, PK parameters such as absorption rate constant (k_a_); elimination rate constant (K_e_); CL, and half-life (t_1/2_); and V_d_, C_max,_ and T_max_ can be determined. This process relies on differential equations, which are resolved using computational software. By solving these equations, drug concentration-time functions are obtained [46,48]. Although more complex than NCA, compartmental PK analyses offer many advantages by providing a deeper understanding and prediction of PK behaviors vis-à-vis variable dosing regimens and systems, which become especially valuable in the later stages of drug development and for precision medications. For example, compartmental models enable population PK analyses, allowing the incorporation of patient-specific factors or covariates that is not possible by using NCA [44]. Therefore, compartmental PK analysis is necessary for the prediction of PK properties of a drug in different settings.

Physiologically based pharmacokinetic (PBPK) models, on the other hand, could be much more complex by integrating physiological parameters (e.g., blood flow, organ sizes) with tissue drug transport, binding, and metabolism characteristics to describe and predict drug concentrations in blood or other tissues [49,50]. Although the PBPK models have some advantages in using in vitro or animal data to predict human PK and understanding the effects of drug regimens or physiological factors on the PK [51,52], their sophisticated nature and the large number of variables or parameters, especially “non-measured” factors or constants, generate some concerns about the validity in fitting and prediction with limited experimental data in hand. While computational tools are broadly available for PBPK modeling, substantial training is generally required, and caution is advised to build and implement proper PBPK models, especially for further integration with PD models. In addition, the route of administration (e.g., intravenous versus subcutaneous) can substantially influence PK characteristics such as absorption rate, bioavailability, and time to peak concentration, yet these processes can be consistently captured using NCA, compartmental, or PBPK models [53,54,55].

In oncology, these PK models have been applied to small-molecule chemotherapies and targeted therapies as well as protein or monoclonal antibody (mAb) agents. For example, a two-compartment model was successfully used to describe the PK of the tyrosine kinase inhibitor dacomitinib and quantify the impact of co-administration of a proton pump inhibitor on its absorption [56]. The PK properties of pembrolizumab, an immune checkpoint–blocking monoclonal antibody, were characterized through a two-compartment analysis of pooled data from various clinical trials, revealing no significant covariate effects and validating the dosing regimen of 2 mg/kg every three weeks, and this dosage was approved for treating inoperable or metastatic melanoma and non-small cell lung cancer (NSCLC) through targeting the immune checkpoint protein, programmed death-ligand 1 (PD-L1) [57]. However, pembrolizumab dosing was updated to a flat 200 mg dose, replacing the earlier 2 mg/kg body weight-based regimen, to improve patient compliance and simplify dosing [58]. An NCA was conducted in a study on the use of trabectedin as a first-line treatment for elderly patients (aged ≥ 70 years) with metastatic soft tissue sarcoma, and the results revealed that trabectedin clearance in the elderly patients was similar to that observed in younger patients, suggesting that trabectedin metabolism is not significantly altered by age [59]. In a comparative study of first-line antitubercular drugs, NCA was used to calculate various PK parameters, including C_max_, T_max_, area under the curve (AUC), t_1/2_, and CL, and to perform comparisons among the drugs [60]. Recently, a whole-body PBPK model was established for the Epidermal Growth Factor Receptor-Tyrosine Kinase Inhibitor (EGFR-TKI) osimertinib to capture heterogeneous tissue distribution and target engagement in NSCLC. By integrating nonlinear PK processes with EGFR-binding dynamics and validating predictions against microdosed [^11^C]-osimertinib PET imaging and clinical PK profiles, the model successfully reproduced concentration–time curves across tumor and normal tissues within two-fold of observed data. This PBPK framework highlights the value of advanced mechanistic modeling in optimizing precision dosing strategies and guiding the development of next-generation TKIs [61]. These studies demonstrate how mathematical modeling can translate early PK data into a well-founded clinical trial design and enhance our understanding of anticancer pharmacology.

## 3. Disease Progression Models to Recapitulate Natural Tumor Growth

When building a PK–PD model, one needs to understand the natural progression of the disease before the addition of drug actions. Therefore, modeling disease progression is important for understanding and predicting the pharmacological effects of drugs, including the anticancer efficacy in oncology. Some important features or factors should be taken into consideration for the development of tumor growth models, such as the initial tumor size, growth rate, and inherent cellular characteristics. Table 1 summarizes several disease progression models (unperturbed tumor growth) commonly found in oncology, such as Gompertz, Logistic, and Simeoni models.

Considering cancer cells divide without any limits, an exponential tumor growth model has been developed to describe unperturbed tumor growth of leukemia in mice [67]. Were cancer cells proliferating without any restrictions, every cell within the tumor would consistently progress through the cell cycle and produce two daughter cells at fixed intervals. In this case, the number of cancer cells and thus tumor volume or mass would grow exponentially over time. As shown in Table 1, the exponential model characterizes the cell count, tumor volume, or weight at a given time [62,63,64,65], where Y(t) represents the tumor size at time t, and λ_0_ is the exponential rate of growth. This geometric progression implies that the time required for the tumor to double in size remains constant [64,76]. While the exponential growth model aligns with the early stages of tumor development, it has been observed that the doubling time eventually increases and continues to lengthen as the disease progresses. This can happen if the average cell-cycle duration extends or if dividing cells are lost due to quiescence or death. As tumor mass or volume increases, its growth rate is commonly diminished, likely due to the decrease in space for the cells to expand, as well as supplies of nutrients and oxygen. Therefore, the exponential growth model fails to accurately describe the long-term growth dynamics of tumors, necessitating a shift away from the straightforward concept of unrestricted cell division to more proper consideration of complex biology and better explanation of the observed disease patterns.

As a result, several asymptotically saturable curves have been suggested, among which the Gompertz model (Table 1) stands out as a notable choice. The Gompertz model was originally introduced in the 19th century as a method for calculating life insurance values by understanding how human mortality rates change with age [77,78], and it was later employed by Anna Laird to explain tumor growth dynamics [19,64]. In this sigmoid-shaped model, the inflection point is reached when the tumor volume attains 37% of its maximum. Beyond this point, the growth rate declines exponentially.

Another model that emerged during the same period is the logistic model (Table 1), also showing a sigmoidal shape, which was initially used to describe population dynamics in 19th century [64,79]. Since then, it has become a cornerstone of biomathematics and has been effectively used to model a wide variety of biological phenomena, from bacterial populations to algae and mammals [63,64]. While the Gompertz equation models a growth rate that decreases exponentially, the logistic equation assumes a linear decline in growth rate as the population size increases and eventually stops. And once the tumor reaches 50% of its final size, the growth rate decreases linearly in relation to the tumor size [63,64,65,70,71]. As shown in Table 1, the Gompertz model tumor growth curve reaches a plateau faster than the Logistic model, although showing a similar shape. This is because the Logistic model primarily considers spatial constraints on tumor growth, whereas the Gompertz model incorporates not only spatial limitations but also other critical factors such as nutrient availability that may influence tumor progression. Reflected in the curve, the Gompertz model assumes a growth rate decreases once the tumor reaches 37% of its maximum volume, whereas the Logistic model assumes that the growth rate declines linearly with tumor size reaches half of its maximum volume.

The Bertalanffy model (Table 1) operates on the assumption that growth is the result of a balance between cell proliferation and cell death [74], whereas it has received considerably less attention. In this model, cell proliferation is proportional to the surface area (with a growth constant α), while the loss of tumor mass due to cell death is proportional to the tumor volume (with a natural loss constant β). The theoretical basis of this model is that the tumor is spherical in shape with volume Y(t), and its surface area scales as Y(t)^2/3^. With further assumption that tumor growth is limited by nutrients and/or oxygen which enter through the surface, the tumor growth rate would be proportional to its surface area, that is, Y(t)^2/3^ [64,80,81]. The Bertalanffy model also exhibits sigmoidal shape and converges to a fixed volume over time when the growth and loss reach balance. The remarkable aspects of the Bertalanffy equation are the inclusion of biologically meaningful parameters in its derivation and accurate fitting of experimental tumor growth curves.

These empirical models face the challenge of accurately estimating the plateau phase because, for ethical reasons, mice are often euthanized when tumor sizes reach a certain threshold. This typically happens before the plateau phase is observed. Model selection is thus guided by parsimony principles, aiming to achieve a sufficient fitting of observed data with fewer parameters possible [3]. Therefore, without effective drug treatment, net tumor growth, which is the result of the balance between growth and natural cell death, most commonly uses exponential function [82] and its derivatives (Table 1), such as exponential growth limited by tumor volume or Yamazaki model [75,83], and a blend of linear and exponential functions or Simeoni and Koch models [20,34,84]. In particular, the Simeoni model permits the tumor growth function to alternate between exponential and linear growth in the absence of a plateau phase [20]. In this case, tumor growth is anticipated to begin exponentially and then transition to a linear phase before plateauing. As experimental data do not show a plateau phase, the Simeoni model explains the exponential and linear growth components, where λ_0_ and λ_1_ are the parameters characterizing the exponential and linear rate of growth (Table 1), and the shape parameter *Ψ* which controls the nonlinearity and smoothness of the tumor growth function is usually fixed at 20 as this number represents a proper turning point for the two distinct phases. The Koch model [34] is another widely recognized tumor growth model that was modified from the Simeoni model to allow a smooth transition between exponential and linear growth (Table 1). Another derivative from the exponential model is the Yamazaki model [75] that includes first order growth and the inhibition of tumor growth by its own tumor size. In Yamazaki model equation (Table 1), Y(t) represents the tumor size, K_in_ is the first-order tumor growth rate constant (hours^−1^), TG_50_ is the tumor size that inhibits 50% of the growth rate which is based on the physiological mechanisms where local factors may constrain tumor growth, and K_out_ is the first-order tumor loss rate constant (hours^−1^).

It should be noted that most of these tumor growth models have been developed and validated primarily in solid tumor contexts, where tumor burden can be directly measured as volume or mass (e.g., in xenograft studies). By contrast, for non-solid tumors such as leukemias, defining tumor size is inherently more difficult, and volumetric models are generally not applicable. In such cases, simple exponential-type growth functions have occasionally been used with surrogate markers (e.g., circulating leukemic cell counts), but overall, disease progression models are less established in hematological malignancies [85,86]. Moreover, the applicability of each model may also depend on the clinical stage: exponential growth better describes early unchecked proliferation, whereas Gompertz, Logistic, and Bertalanffy models capture growth deceleration observed in more advanced stages. Simeoni, Koch, and Yamazaki models are particularly useful in preclinical xenograft treatment-response settings, where both tumor shrinkage and regrowth can be captured. In the subsequent section, we further discuss the applications of these disease progression models in greater detail, including their integration with drug effect modeling.

## 4. PD Models for PK-PD Modeling of Anticancer Monotherapy

**Figure 1:**
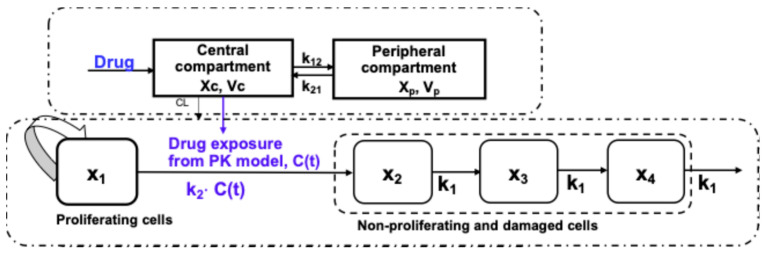
Illustration of an integrated PK–PD model. PK: two-compartment model; PD: cell distribution model. *C(t)*, plasma concentration of drug at time t; *CL*, drug clearance; *k*_1_, transient rate constant; *k*_2_, potency of the drug; *k*_12_ or *k*_21_, apparent first-order intercompartmental transfer rate constants; *V*_c_ or *V*_p_, volume of distribution in central or peripheral compartment; *x*_1_, the mass of the proliferating cells; *x*_2_, *x*_3_, *x*_4_, the mass of non-proliferating cells at different damage states; *X*_c_ or *X*_p_, Drug amount in central or peripheral compartment.

As an anticancer drug is administered to suppress tumor growth through its pharmacological actions within the biological system, an integrated PK–PD model provides a comprehensive understanding of quantitative relationship between drug exposure and tumor or biomarker response over time. The established PK–PD model shall allow one to quantitatively assess how drug concentrations change over time and influence therapeutic outcomes, following drug administration, and thus optimize treatment regimens at different scenarios. Some common PD models used for integrated PK–PD modeling of anticancer drug monotherapy are summarized in Table 2, and a specific two-compartment PK model linked to a transient compartment PD model is depicted in Figure 1.

### 4.1. Indirect Response Models

Many PD models attempt to account for treatment effects using mechanistic or semi-mechanistic models. The indirect response (IDR) models [87], widely used in PK–PD modeling, include four basic models to characterize drug effects, in which the drugs may control the input (production; represented by constant K_in_) or dissipation (loss; represented by constant K_out_) of particular outcomes (e.g., tumor growth inhibition (TGI) or changes in biomarkers) through inhibition or stimulation mechanisms. The IDR models employ the Hill equation function to describe the measurable changes with drug concentrations over time until a maximum is reached. Linked to proper PK models, the IDR models may be used to classify dose–response data that demonstrate either increased (stimulation of K_in_ or inhibition of K_out_) or diminished (inhibition of K_in_ or stimulation of K_out_) responses over time. On the other hand, knowledge of the mechanistic action of a drug (e.g., agonist or antagonist of targets controlling K_in_ or K_out_) is helpful to select the right IDR model for PK-PD data analyses and predictions. Therefore, the IDR models are applicable to a wide range of drugs that exert pharmacological effects through either inhibiting or activating specific factors, such as histamine antagonists, hypoglycemic agents, angiotensin-converting enzyme inhibitors, and dopamine antagonists [88,89,90,91,92,93].

**Table 2:** Common PK-PD models for monotherapy in oncology.

Model	Mathematical Equation	References
IDR		
Inhibition of K_in_	$\frac{dR}{dt}=K_{in}\times \left(1-\frac{E_{max}\times C^{\gamma }}{{EC}_{50}^{\gamma }+C^{\gamma }}\right)-K_{out}\times R(t)$	[75,87]
	$\frac{dx}{dt}=K_{in}^{\prime }\times \left(1-\frac{x\left(t\right)}{{TG}_{50}+x\left(t\right)}\right)\times \left(1-\frac{E_{max}\cdot C^{\gamma }}{{EC}_{50}^{\gamma }+C^{\gamma }}\right)\times x\left(t\right)-K_{out}^{\prime }\times x(t)$	
	$x\left(t\right)$—the tumor mass at time t R—Biomarker γ—Hill coefficient $K_{in}$—the zero-order formation rate constant (h^−1^) $K_{out}$—the first-order degradation rate constant (h^−1^) $K_{in}^{\prime }$—the first-order tumor growth rate constant (h^−1^) $K_{out}^{\prime }$—the first-order tumor loss rate constant (h^−1^) TG_50_—the tumor mass that inhibits 50% of the tumor growth rate	
Stimulation of K_out_	-	-
**Signal distribution**	$\frac{dx}{dt}=k_{g}\times x\left(t\right)-K_{4}\times x\left(t\right)$ $,\;x\left(0\right)=w0$	[82]
	$\frac{dK_{1}}{dt}=(\frac{K_{max}\times C}{{IC}_{50}+C}-K_{1})/\tau$	
	$\frac{dK_{2}}{dt}=(K_{1}-K_{2})/\tau$	
	$\frac{dK_{3}}{dt}=(K_{2}-K_{3})/\tau$	
	$\frac{dK_{4}}{dt}=(K_{3}-K_{4})/\tau$	
	$K_{1}\left(0\right)=K_{2}\left(0\right)=K_{3}\left(0\right)=K_{4}\left(0\right)=0;w\left(t\right)=x$ $x\left(t\right)$—the tumor mass at time t $k_{g}$—the net tumor growth constant $K_{max}$—the maximal cell kill rate ${EC}_{50}$—the half maximal effective concentration $K_{1},\;K_{2},\;K_{3},\;K_{4}$—the cell kill rate constants in different transit compartments C—the plasma concentration of drug τ—the transit time $w\left(t\right)-$the total tumor mass at time t	
**Cell distribution**		
Simeoni	$\frac{dx_{1}}{dt}=\frac{\lambda_{0}\times x_{1}\left(t\right)}{[1+(\frac{\lambda_{0}}{\lambda_{1}}\times {w(t))}^{\psi }{]}^{\;\frac{1}{\psi }}}-k_{2}\times C\left(t\right)\times x_{1}(t)$	[20]
	$\frac{dx_{2}}{dt}=k_{2}\times C\left(t\right)\times x_{1}\left(t\right)-k_{1}\times x_{2}(t)$	
	$\frac{dx_{3}}{dt}=k_{1}\times x_{2}(t)-k_{1}\times x_{3}(t)$	
	$\frac{dx_{4}}{dt}=k_{1}\times x_{3}(t)-k_{1}\times x_{4}(t)$	
	$w\left(t\right)=x_{1}\left(t\right)+x_{2}\left(t\right)+x_{3}\left(t\right)+x_{4}(t)$	
	$x_{1}\left(0\right)=w_{0};\;x_{2}\left(0\right)=x_{3}\left(0\right)=x_{4}\left(0\right)=0$ k_1_—the transient rate constant k_2_—the potency of the drug λ_0_—the exponential rate of tumor growth λ_1_—the linear rate of tumor growth ψ—the shape parameter controls the nonlinearity and smoothness of the tumor growth function x_1_—the mass of the proliferating cells x_2_, x_3_, x_4_—the mass of the non-proliferating cells at different damage stages $w\left(t\right)$—the total tumor mass at time t $C\left(t\right)$—the plasma concentration of the drug at time t	
Koch	$\frac{dx_{1}}{dt}=\frac{2\times \lambda_{0}\times \lambda_{1}\times {x_{1}(t)}^{2}}{\left(\lambda_{1}+2\times \lambda_{0}\times x_{1}\left(t\right)\right)\times w(t)}-k_{2}\times C\left(t\right)\times x_{1}(t)$	[34]
	(All other equations remain the same as Simeoni’s)	
Tumor burden	$\frac{dV_{1}}{dt}=\left(k_{g}-k_{1}\times \frac{E_{max}\times C\left(t\right)}{{EC}_{50}+C\left(t\right)}\right)\times V_{1}{\left(t\right)}^{\frac{2}{3}}$	[94]
	$\frac{dV_{2}}{dt}=k_{1}\times (\frac{E_{max}\times C(t)}{{EC}_{50}+C(t)}\times V_{1}{\left(t\right)}^{\frac{2}{3}}-V_{2}{\left(t\right)}^{\frac{2}{3}})$	
	$\frac{dV_{3}}{dt}=k_{1}\times (V_{2}{\left(t\right)}^{\frac{2}{3}}-V_{3}{\left(t\right)}^{\frac{2}{3}})$	
	$TV\left(t\right)=V_{1}\left(t\right)+V_{2}\left(t\right)+V_{3}\left(t\right)$	
	$TV\left(0\right)=V_{1}\left(0\right);V_{2}\left(0\right)=V_{3}\left(0\right)=0$ $V_{1}\left(t\right)$—the volume of dividing cells at time t $V_{2}\left(t\right),$ $V_{3}\left(t\right)-$the volume of damaged cells at different stages at time t $TV\left(t\right)$—the total tumor volume at time t $k_{g}$—the net tumor growth constant k_1_—the transient rate constant $E_{max}$—the maximal effect ${EC}_{50}$—the half maximal effective concentration $C\left(t\right)$—the plasma concentration of drug at time t	
**Immune checkpoint inhibition**	$\Omega_{C}=\frac{C}{\frac{C}{\left[1-\frac{P}{P+\sum_{j}\left(T_{3,j}+T_{4,j}\right)}\right]}+\sum_{j}\left(T_{3,j}+T_{4,j}\right)}$	[95]
	$\Omega_{C}$—the overall portion of inhibited effector memory T cell and effector T cells;	
	C—the number of cancer cells;	
	P—the concentration of free PD-1 inhibitor in blood	
	$T_{i,j}$—the number of T cells in the i th differentiation compartment that have undergone j divisions	

In oncology, the IDR models are also valuable to perform PK–PD modeling of any anticancer drugs that “indirectly” inhibit tumor growth by acting on respective targets. And the modeling framework is especially relevant for drugs exhibiting a delayed onset of action, allowing researchers to recapitulate the dynamic interplay between drug effects and biomarker responses over time. For instance, IDR models were used to characterize the relationship between the concentrations of c-mesenchymal–epithelial transition factor (cMet) inhibitor PF02341066, a targeted therapy candidate small molecule, and the inhibition of cMet phosphorylation (biomarker), as well as TGI (pharmacological response) in human tumor xenograft (GTL16 gastric carcinoma) mouse models and then further assess the relationship between biomarker and TGI efficacy [75]. Both IDR models in this study involve the inhibition of K_in_ (Table 2) The IDR model for biomarker described a delayed onset of cMet phosphorylation following PF02341066 administration, suggesting a rate-limiting distribution from plasma into tumor. By fitting the biomarker IDR model to the time-course data, the EC90 (167 ng/mL) was determined for the inhibition of cMet phosphorylation. To assess the relationship between cMet phosphorylation inhibition and antitumor efficacy, the IDR model was again applied to this tumor growth inhibition data, allowing the investigators to estimate the EC50 value (213 ng/mL) for the TGI. Therefore, the EC90 for cMet phosphorylation inhibition was found to align with the EC50 for tumor growth inhibition, indicating that achieving near-complete suppression of cMet phosphorylation (>90%) is necessary to obtain substantial tumor growth reduction (>50%). The use of IDR models in this study was able to describe the relationships between the PK of PF02341066, the inhibition of cMet phosphorylation, and final TGI activity.

Another example is the use of IDR model for PK–PD modeling of TAK-441, a selective Smoothened (Smo) antagonist in the hedgehog signaling pathway [83]. By characterizing the changes in glioma-associated oncogene 1 (Gli1) mRNA in tumor (biomarker) with an IDR model (inhibition of K_in_, Table 2), the researchers were able to find that over 94% inhibition of tumoral Gli1 mRNA levels would be required to sufficiently inhibit (>90%) hedgehog-related tumor growth in mice bearing xenografts of human pancreatic tumors suggesting that tumoral Gli1 mRNA level could be a useful biomarker for predicting the antitumor effect of hedgehog inhibitors [83]. Similarly, the IDR model with the inhibition of K_in_ has been applied to PK–PD modeling of some other targeted therapeutic agents, such as GDC-0941 (a phosphoinositide 3-kinase inhibitor) [96], crizotinib (a dual inhibitor to anaplastic lymphoma kinase and the hepatocyte growth factor receptor) [97], and ONO-7579 (a pan-tropomyosin-related-kinase inhibitor) [25], as the TGI effects of targeted therapies often exhibit a delayed response following interactions with their molecular targets.

Although it is possible that such cases could exist and in the presence of many variants of IDR models [12], we were surprisingly unable to find any articles reporting the use of an IDR model with the stimulation of K_out_ for the PK–PD modeling of anticancer drugs, such as those agents known to mechanistically induce apoptosis. One possible reason is that, unlike the inhibition of K_in_, where the biomarkers are directly related to the agents and relatively easy to measure, stimulation of K_out_—referring to drug-induced effects such as apoptosis, necrosis, or other forms of programmed cell death in tumor cells—might only represent partial drug effects to recapitulate the overall TGI.

### 4.2. Signal Distribution Models

An alternative to the IDR model is the signal distribution model (SDM) (Table 2), which was initially developed to capture the effects of chemotherapeutic methotrexate on cancer cell growth in vitro [82] and subsequently applied in vivo [98,99]. SDM is commonly used to characterize the signaling process within a biological system by using transit compartments where there is a delayed onset of any phenomena observed. In the context of cancer therapy, this model can be applied to understand how signaling pathways are modulated upon drug-target interactions to explain the delay between drug dosing and TGI, where *x* represents the tumor size, τ denotes the transit rate constant, and k_g_ is a tumor growth function (Table 2) [82,99,100]. For example, the SDM was successfully applied to PF04942847, a small-molecule inhibitor to heat shock protein 90 (HSP90), given a temporal delay between plasma concentrations of PF04942847 and the inhibition of its downstream protein, protein kinase B (PKB/AKT) in a human breast cancer MDA-MB-231 xenograft tumor mouse model [98]. TGI was effectively characterized using the SDM, with plasma concentrations serving as the driving force. Furthermore, the integration of AKT degradation dynamics with tumor growth inhibition indicated that greater than 30% degradation of AKT was required to achieve more than 50% inhibition of tumor growth [98]. In addition, one study compared the SDM with the following Simeoni model for the analysis of paclitaxel effects on the progression of colon cancer xenograft tumors in mice [99], showing that both models were able to describe the dose-dependent therapeutic responses of Colon-26 tumors, although they are mechanistically distinct.

### 4.3. Cell Distribution Models

The Simeoni model [20] describes how anticancer drugs drive proliferating tumor cells into a non-proliferative state before they eventually die. By incorporating transit compartments to represent proliferating, damaged, and dead cells, this model explains the delayed TGI following drug exposure and is therefore categorized as a cell distribution model (CDM). The PD parameters consist of those related to natural or empirical tumor growth characteristics, drug potency, and tumor cell death kinetics. As shown in Table 2, k_2_ in the equations is a parameter representing the drug efficacy in killing the tumor, k_1_ is the rate of cell death caused by the drug, w(t) is the total tumor weight, and C(t) is the drug concentration at time t. While three compartments of nonproliferating or damaged cells are common to recapitulate TGI, the number of transit compartments can be modified accordingly. The Simeoni CDM seems more closely to incorporate the mechanism of anticancer actions and separate drug-related parameters from natural tumor growth dynamics, and thus it has become a popular PD model in oncology. Indeed, the Simeoni model accurately fitted experimental data for various anticancer drugs at different doses and schedules in xenograft tumor mouse models, including paclitaxel, 5-fluorouracil, and irinotecan [20,101], and provided reliable parameter estimates. To support oncology trials and clinical practice, some secondary parameters like time efficacy index and threshold concentration for tumor eradication were also introduced, and subsequent studies showed that the threshold concentrations predicted from preclinical xenograft mouse studies matched the active doses observed in humans for several commercially available chemotherapy drugs, including cisplatin, pemetrexed, gemcitabine, doxorubicin, vinblastine, and docetaxel [102,103].

The Simeoni CDM was also applied to targeted therapy in oncology. One study demonstrated that the antitumor effects of the multi-kinase inhibitor sorafenib, the Feline McDonough Sarcoma-like tyrosine kinase 3 (FLT3) inhibitor quizartinib, and the bifunctional FLT3 plus cyclin-dependent kinases 4 and 6 inhibitor AMG925 in subcutaneous and orthotopic mouse models of acute myeloid leukemia could be described well by the Simeoni model [104]. Interestingly, in this study, the effects of these targeted therapeutics on downstream targets, such as signal transducer and activator of transcription 3 and retinoblastoma protein phosphorylation, were fitted using the IDR model (inhibition of K_in_) [104]. This suggests that, in the context of targeted therapy research, establishing a composite model to relate the blood PK of a drug to its effects on corresponding molecular targets and the resulting TGI is feasible and informative. As another example, a recent study showed that the Simeoni model successfully characterized the PK–PD of siremadlin (a small-molecule inhibitor to the mouse double minute 2 homolog protein) and trametinib (a small-molecule inhibitor to mitogen-activated protein kinase kinase) in a melanoma mouse xenograft model [105].

The Koch model (Table 2) was derived from the Simeoni model, and it is featured by a smooth transition from exponential to linear growth phases for the tumor growth while retaining the principle of CDM and using the transit compartments to describe the effects of anticancer drugs [34]. The Koch CDM was extended from monotherapy to combination therapy [34]. The latter will be discussed in the following section.

Another derivative of the CDM, namely the tumor burden model (Table 2), was initially developed to characterize the antitumor effects of trastuzumab-mertansine (T-DM1), an antibody–drug conjugate for the treatment of human epidermal growth factor receptor 2 (HER2)-positive breast cancer [94]. As the PK of T-DM1 in mouse models were described well by a two-compartment model, tumor volume (TV) rather than tumor mass (*x*) was used to depict tumor burden and in response to T-DM1 therapy in animal models of HER2-positive breast cancer. A cell-cycle-phase nonspecific tumor cell kill model, which incorporated transit compartments, effectively represented the characteristics of tumor growth and the action of T-DM1. The tumor cells were categorized into two groups: drug-insensitive cells that grow at a constant rate, and drug-sensitive cells that ultimately die following progression of damage and cessation of replication. The total tumor volume is the sum of the insensitive cells and the sensitive cells at various stages of death. Through this model, the researchers discovered that tumor response was linked to the ratio of exposure to a concentration required for tumor stasis (tumor-static concentration, TSC). Therefore, early clinical trials were designed to target exceeding the TSC (30.2 mg/L) in many patients [106,107]. This tumor burden model and the calculation of TSC were proven useful for studying antibody–drug conjugates in oncology, including trastuzumab deruxtecan that targets HER2 and monomethyl auristatin E (MMAE) that targets mesothelin, respectively [108,109,110].

### 4.4. Other PD Models for Integrated PK-PD Modeling of Anticancer Drugs

The advancements in computational capabilities have led to the development and application of models for anticancer drugs with more complex mechanisms of action, such as immunotherapy drugs. For example, a computational model was developed to incorporate cellular immunity and tumor growth for the analysis of time-dependent PD effects of pembrolizumab towards the identification of response predictors and precision medication [95]. Clinical data from a melanoma patient were used for calibration, and this complex model (Table 2) was able to accurately capture the patient’s atypical disease dynamics, including a temporary increase in tumor burden followed by a delayed and long-lasting tumor shrinkage, revealing that lower cytotoxicity of effector cluster of differentiation 8-positive T cells can lead to an accelerated tumor progression. The ratio of T cell reinvigoration to baseline tumor load was shown as a potential predictor of immunotherapy drug response, emphasizing the need for personalized medication to enhance treatment effectiveness. Nevertheless, studies with larger sample sizes are necessary for more accurate predictions.

To further illustrate, immunotherapy modeling presents unique challenges and opportunities compared with conventional cytotoxic or targeted therapies. Unlike small-molecule agents, immune checkpoint inhibitors such as pembrolizumab, nivolumab, and ipilimumab can induce nonlinear and time-dependent response patterns, including pseudo-progression and delayed tumor shrinkage, which are difficult to describe with traditional PK–PD models alone [111,112,113]. Mechanistic frameworks that explicitly incorporate immune cell dynamics (e.g., T cell activation, expansion, and exhaustion), cytokine signaling, and tumor–immune interactions have therefore been increasingly applied [113,114,115]. Similar approaches have also been extended to cellular immunotherapies, such as CAR-T cells, to capture phenomena like cytokine release syndrome and interpatient variability in expansion kinetics [116,117,118]. These examples underscore the need for more mechanistic, systems-based models in immuno-oncology, highlighting both the promise and the complexity of translating PK–PD principles into this therapeutic domain.

On the other hand, while current PD models have demonstrated utilities for quantitative understanding of PK–PD relationships of many anticancer drugs, new concepts and practical models are warranted for the development of new classes or generations of drugs with more complex mechanisms of actions. Most recently, model-informed drug development (MIDD) approaches have become increasingly important in regulatory science and clinical pharmacology, as they integrate various PK and PD models to inform decision-making in drug discovery and development [119,120,121,122]. In parallel, physiologically based PK–PD (PBPK–PD) models have been developed to bridge preclinical and clinical studies, allowing the incorporation of drug–target interactions, disease-related physiological changes, and complex dosing regimens to better predict clinical outcomes [123,124,125]. Furthermore, the emergence of quantitative systems pharmacology (QSP) provides a systems-level modeling framework that combines pharmacology, systems biology, and disease modeling to capture multi-scale drug–disease–patient interactions, thereby enabling more mechanistic and predictive approaches in drug development [126,127,128,129]. These newer modeling paradigms take advantage of advanced computational tools and follow the principles of PK–PD modeling to offer predictive, mechanistic, and translatable insights in a given system that support precision medicine and rational combination therapies in oncology and other therapeutic areas.

Conceptually, these modeling paradigms can be viewed in a hierarchical framework: “practical” PK–PD models often serve as the foundation, providing parsimonious and empirically driven descriptions of exposure–response relationships [128,130]. PBPK–PD and QSP approaches represent more mechanistic, bottom-up frameworks that can encompass simpler PK–PD models as subsystems [131,132]. While practical models are often sufficient for many oncology applications, such as dose optimization or exposure–response analyses in relatively well-characterized drugs, more complex models like QSP may be warranted when the underlying mechanisms are poorly understood, involve multiple interacting pathways, or require the integration of diverse biomarker and clinical datasets [133]. This hierarchical view helps clarify the complementary roles of different modeling approaches, with PK–PD models forming the empirical foundation and PBPK–PD and QSP approaches representing increasingly mechanistic extensions. Together, these approaches highlight a continuum from parsimonious to mechanistic modeling, with model choice guided by the complexity of the biological question and the available data [134,135].

While most of the tumor growth inhibition and PK–PD models can in principle be applied across drug classes, certain usage patterns are more commonly observed. For small-molecule therapies, IDR and signal distribution models have frequently been employed, as they capture delayed pharmacodynamic responses mediated through downstream signaling pathways [25,75,83,96,98,99]. Cell distribution models, such as the Simeoni and Koch frameworks, are widely used in preclinical xenograft studies of cytotoxic chemotherapies [20,101,102,103] but have also been extended to targeted small molecules [104,105]. In contrast, tumor burden models have found particular utility in describing the effects of antibody-based therapies, especially antibody–drug conjugates, where drug-sensitive and insensitive tumor cell populations can be differentiated [94,106,107,108,109,110]. For immuno-oncology agents, including immune checkpoint inhibitors and CAR-T therapies, more mechanistic and systems-based approaches are often required to account for atypical response patterns, such as pseudo-progression, delayed response, and immune-cell expansion kinetics [113,114,115,116,117,118]. These examples highlight that model choice is not rigidly defined by modality, but is influenced by the mechanism of action, the availability of biomarkers, and the biological processes underlying tumor–drug interactions.

## 5. Advanced PD Models for PK-PD Modeling of Combination Therapy and the Determination of Combination Effects

Combination therapies are commonly used and necessary for the treatment of advanced and highly heterogeneous cancer. An integrated PK–PD model provides a useful framework to holistically evaluate drug efficacy related to the exposure to co-administ ered drugs. This approach enables the quantification of individual drug contributions and possible combination effects on the overall pharmacological response, synergistic, additive, or antagonistic effects. In particular, a synergism where the combined effect of two or more drugs is greater than the sum of their individual effects, is highly desired for combination therapy. The synergistic efficacy may result from various mechanisms occurring at both the PK and PD levels, such as the increase in drug exposure and complementary pharmacological actions. Understanding and quantifying synergism is crucial for optimizing combination therapies in cancer treatment, as it can assist in identifying more effective regimens with less side effects. Three advanced PK–PD models for combination therapy are summarized in Table 3, including respective parameters introduced to describe drug–drug interactions.

### 5.1. Koch Model

The Koch Model of combination therapy is to use an interaction parameter (*ψ*) to determine the total influence of a drug combination while the TGI potency of drug A and B is denoted as k_2A_ and k_2B_, respectively [34] (Table 3). This single metric introduced into the Koch Model readily characterizes the nature and intensity of interactions between co-administered drugs, applicable to any form of monotherapy PD models (Table 2), which facilitates the comparison of different drug combinations and choice of models towards improved understanding of drug actions and identification of optimal combination TGI. As the estimated interaction factor ψ value straightforwardly indicates the final combination outcome, e.g., equal (ψ = 1), less (ψ < 1), or greater (ψ > 1) efficacy [33], it was proposed in the original report [34] and then has been used in many studies [36,37,136,137,138,139] to indicate synergism (ψ > 1), additivity (ψ = 1), and antagonism (ψ < 1) which was rather questionable [33] and elaborated further in the following Section 5.3.

Another caveat of the Koch combination therapy PK–PD model is the random assignment of interaction parameter *ψ* (Table 3) to any of the drug co-administered without clear criteria and sufficient evidence, as pointed out recently [33]. Therefore, when the interaction factor ψ is applied on different drug, such as drug A versus drug B, the same set of experimental data will give different modeling or contradictory results, which was well illustrated in a recent study by using doxorubicin and sorafenib as a model combination [33]. This was also noted in an early study on the combination of gemcitabine and birinapant against pancreatic cancer xenograft tumor growth where the estimated ψ was 1.27 when assigned to birinapant, whereas it was 0.944 when assigned to gemcitabine [140], which is hard to explain.

In addition, the confusion remains whether the interaction factor ψ should be applied solely on a PD factor (e.g., ψ × k_2_ or ψ × EC50 or ψ × half-maximal inhibitory concentration (IC50)) [36,139,140,141] to recapitulate the PD interactions, or the overall effect of one drug where dynamic drug concentrations being taken into consideration (e.g., *ψ* × $\frac{E_{max\;}\times \;c}{{EC}_{50}+\;c}$ or ψ × (k_2_ × c_2_(t))) [33,34,37,136,137,138], and thus interpreted accordingly. The latter is generally more practical while the former will benefit from knowledge and integration of PK interactions, if known, into the PK–PD model. Therefore, we thought that the opposite modeling results in the study on gemcitabine and birinapant combination [140] might be due to multiplying only the EC50 of birinapant rather than the whole Hill equation formula by ψ, whereas in the other case the ψ was actually applied on the overall gemcitabine effects (i.e., ψ × (k_gemcitabine_ × c_gemcitabine_)) instead of the cell killing potency alone (i.e., ψ × k_gemcitabine_) [140].

### 5.2. Terranova Model

Assuming an absence of interactions between the drugs co-administered, a zero-interaction model was initially established [22] by simply combining TGI models of individual drugs [20]. Predictions from this zero-interaction model are then compared to the experimental TGI to validate an “additivity” of the combined drugs. By contrast, derivations of the experimental TGI data from the model predictions forecast potentially synergistic or antagonistic interactions [22].

By introducing an interaction term $\nu_{ij\;}$ (Table 3) into this zero-interaction model, which was assumed to be proportional to individual drug concentrations through an interaction parameter γ, as well as the weight of proliferating cells, as shown below,

$$\nu_{ij}=\left\{\begin{array}{c} \gamma \;{\times \;C}_{A}\left(t\right)\;\times \;\left(t\right)\;{\times \;x}_{00},\;i=j=1\\ 0,\;\mathrm{otherwise} \end{array}\right.$$

a new complete PK–PD model named the Terranova Model (Table 3) was developed for combination therapy in oncology [35]. This model was successfully applied to six experiments involving multiple types of human carcinoma cell lines (ovarian, colon, and pancreatic) derived xenograft mouse models and a number of new compounds or marketed drugs, including irinotecan, 5-fluorouracil, cisplatin, and gemcitabine [35]. Its predictive power was validated by using the estimated parameters from one combination experiment to predict the outcome of a new combination treatment with the same drugs. Specifically, the model was used to predict the outcome of a new combination therapy involving the same drugs but administered with different schedules or doses, and these predictions were compared with actual collected data. The predicted regimens were revealed as close to those used in the model fitting, which would be valid within a reasonable dose range. However, if the doses and intervals in the combination therapy differ largely from those used for the individual treatments, the nature and extent of the drug interactions will likely become largely variable. Indeed, an interaction parameter γ and its derived interaction term $\nu_{ij}$ should be unique for a particular combination, and thus its utility to predict the outcomes of distinct combinations is a dilemma.

Like the Koch combination therapy PK–PD model, the Terranova Model (Table 3) is applicable to different PK–PD monotherapy models (Table 2) for co-administered drugs with the same or distinct mechanisms of actions, even without the detailed information about the drug actions or specific interactions. Rather, the Terranova Model is more complex than the Koch Model in that the tumor cells are assumed to be simultaneously damaged by individual drugs and sequentially destroyed by other drugs, whereas the interaction effect ($\gamma {\;\times \;C}_{A}\;{\times \;C}_{B}$) occurs only at the initial step [35]. In addition, similar as the interaction parameter ψ in the Koch combination therapy PK–PD model, the combination parameter γ within the Terranova Model (Table 3) was proposed for the determination of additivity (equal or close to zero), antagonism (less than zero), or synergism (greater than zero) of the co-administered drugs to achieve TGI, besides signifying the absence or presence of negative or positive drug interactions [35]. Likewise, the validity of using the combination parameter γ to indicate pharmacological synergism beyond a potentiated or improved effect is questionable, as aforementioned and elaborated in the following section.

### 5.3. Choi-Yu Model

**Figure 2:**
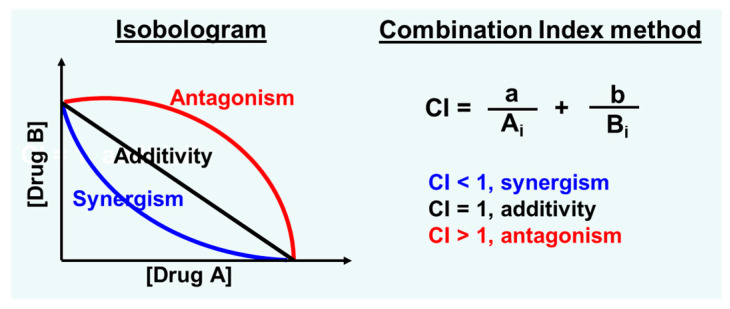
Commonly used isobologram and combination index (CI) method for the determination of combination effects. A_i_ and B_i_ represent the equivalent dose of the drug A and B, respectively, to achieve a specific efficacy (e.g., 50% TGI) as administered alone, while a and b are respective dose of drug A and B in the combination.

Pharmacological or toxicological synergism, antagonism, or additivity between co-administered drugs should be critically defined by using a valid algorithm, such as the classic isobologram or Loewe approach and the commonly used combination index (CI) or Chou–Talalay method (Figure 2), which are actually equivalent [142,143,144]. It is noteworthy that a simple comparison between the effects of combined drugs A and B (E_A+B_) and individual ones (E_A_ or E_B_), i.e., E_A+B_ > E_A_ or E_A+B_ > E_B_, does not necessarily indicate a combination synergy that is conceptually different from and cannot be mixed up with an enhancement or potentiation observed in a study [145,146]. This is also true for the determination of antagonistic effects.

By definition, a synergism occurs when the observed effect of combined drugs is greater than the sum of individual drug effects, i.e., E_A+B_ > E_A_ + E_B_. However, synergism cannot be determined if the sum of individual drug effects examined is already equivalent to or greater than the maximal effect (E_max_), i.e., E_A_ + E_B_ ≥ E_max_, because no treatment, including combination therapy, can exceed the maximal effect (e.g., 100% TGI in oncology). Most importantly, the synergistic nature and, especially, the extent of combination effect of one specific combination regimen are not predictive of those of different combinations, which are usually distinct from each other [147,148,149,150]. Therefore, the interaction factor ψ in the Koch combination therapy PK-PD model [34] and combination parameter γ in the Terranova Model [35] (Table 3) can only indicate the combination effect (e.g., synergism) of a specific drug combination studied experimentally, and literally they cannot be assumed as unchanged and thus used for the prediction of the efficacy of different combinations [33]. Were the interaction factor ψ or combination parameter γ value assumed as unchanged to enable the simulations of TGI of different combinations, the same ψ or γ value would have already pre-defined the nature and degrees of distinct combination effects, either synergism, antagonism, or additivity, which is obviously controversial.

A new generic PK–PD model of combination therapy was thus developed by the Yu lab, enabling the use of proper approaches, such as Chou–Talalay method, for the evaluation of combination synergism [33]. Considering that one drug would affect the overall efficacy of another drug co-administered, and vice versa, either due to PK or PD interactions or both, contribution factors were introduced in the Choi-Yu Model to qualitatively and quantitatively recapitulate the actual contributions of individual drugs (e.g., α and β for combined drug A and B, respectively) to the overall combination effect (Table 3). The contribution factors can also be viewed as interaction parameters to indicate the nature and extent of one drug affected by other co-administered drugs. For instance, a value less than 1.0 (e.g., α = 0.80) indicates that drug A contributes less to the final combination drug efficacy, presumably due to the reduction of drug A monotherapy efficacy by the co-administered drugs. By contrast, a value greater than 1.0 (e.g., β = 1.2) signifies that drug B contributes more to the overall combination efficacy, being a potentiation of drug B monotherapy efficacy by the co-administered drugs. Assuming the nature and degrees of interactions between co-administered drugs retain under different dosing regimens, the values of individual contribution factors (α and β, etc.) estimated from an experimental combination would remain the same for other combinations, which enables the simulation of efficacy for untested combinations and most importantly, the calculation of CI values (Chou–Talalay method) for the determination of possible synergism, additivity, and antagonism [33].

The Choi-Yu combination therapy PK–PD model [33] was employed to evaluate doxorubicin plus sorafenib combination therapy in xenograft mouse models [151]. The contribution factor of sorafenib was revealed to be greater than 1.0 (β = 1.62), exerting a considerably greater influence on TGI by the combined drugs, whereas doxorubicin efficacy was dampened (α = 0.644) in combination therapy. Through PK–PD modeling, new dosage combination was readily identified to achieve optimal TGI, consistent with the greatest extent of synergism that was manifested by the calculated CI value [33], which warrants further experimental validation. Most excitingly, the modeling and simulation findings based on the Choi-Yu combination PK–PD model [33] elucidate the “inexplicable” clinical observations [152,153] that the addition of sorafenib to doxorubicin therapy markedly enhanced the therapeutic efficacy, whereas the incorporation of doxorubicin to sorafenib treatment did not improve the therapeutic outcome, highlighting the utilities of proper PK–PD modeling in understanding of experimental observations, preclusion of nonbeneficial combinations and related experiments, and identification of optimal combination for trials and therapy.

Compared with the Koch Model (Table 3), in which the interaction factor may be applied to any drugs co-administered to offer variable or opposite results that are hard to explain, the Choi-Yu Model provides a more definitive modeling or simulation result for one specific combination [33]. Different from the use of a single interaction factor *ψ* or interaction parameter γ, which can lead to an oversimplified and potentially inaccurate evaluation of combination synergism, the apparent contribution factors of individual drugs (α and β, etc.) in the Choi-Yu Model offer a more intuitive interpretation of possible pharmacological interactions and apparent contributions to final outcomes and enables a rigorous evaluation of combination effect by using a valid approach, such as the Chou–Talalay CI method. Nevertheless, the contribution or interaction factors of individual drugs (α and β, etc.) might not necessarily remain the same in different combinations, and this model warrants further validation or possible refinement.

## 6. Conclusions and Perspectives

PK–PD modeling has emerged as a powerful tool in drug development, especially for the exploration of optimal combination therapy for the treatment of highly heterogeneous cancer disease. An integrated PK–PD model enables the quantitative characterization of the mechanistic drug exposure–response relationship, which is needed for the identification of optimal dosing regimens subjected to clinical investigations or the practice of precision medication. Many PK–PD models have been established in oncology for studies on mono- and combination therapies. Although a single interaction factor or parameter introduced to recapitulate the interaction of drugs co-administered can signify the nature and degree of change in efficacy of one specific combination, its utility for the prediction of different combinations, and especially the determination of combination synergism, is problematic. By contrast, the Choi-Yu model, developed recently by considering apparent contributions from individual drugs to overall combination outcome, is plausible, as it assumes that drugs co-administered may interact with each other and identifies correct algorithm, such as the CI method, that should be used, along with experimental or PK–PD modeling data, to achieve a rigorous and reliable evaluation of combination synergism.

Most of the PK–PD modeling approaches reviewed in this article have been developed and validated primarily in xenograft experiments. This is not surprising, as xenograft systems provide a relatively well-controlled and reproducible platform compared with more variable clinical trials, allowing for systematic evaluation of drug exposure–response relationships and facilitating model development [20,35]. However, while xenografts have greatly contributed to the maturation of PK–PD modeling in oncology, their inherent limitations in mimicking the human tumor microenvironment and predicting clinical outcomes should be acknowledged.

In particular, translating these models into the clinical setting remains a formidable challenge. Clinical trials often face sparse data availability, since patients provide limited PK samples and repeated tumor biopsies for PD biomarkers are rarely feasible [154]. Tumor heterogeneity, both across patients and within individual tumors, further complicates model predictions, as real-world cancers display far greater genetic and microenvironmental diversity than xenografts [155,156]. Additionally, biomarker availability in patients is restricted, making it necessary to rely on surrogate or non-invasive measures such as circulating tumor DNA or imaging-based endpoints, which themselves require extensive validation [157,158]. Finally, in terms of real-world application, model-informed drug development increasingly leverages population PK–PD modeling and the integration of clinical trial and electronic health record data. While such approaches hold great promise for bridging preclinical and clinical domains, challenges related to data quality, standardization, and confounding factors continue to limit their widespread implementation [158,159].

As the complexity of drug combinations continues to rise, it is vital to refine current PK–PD models or develop new tools to characterize drug exposure–response relationships and possible drug–drug interactions or individual drug contributions for the prediction of optimal dosing regimens to achieve maximal therapeutic efficacy with the strongest synergism. In addition, the identification and utilization of valid PD biomarkers, development of mechanism-based PD models, and adoption of newly developed machine learning or artificial intelligence technologies should undoubtedly advance PK–PD research and make PK–PD modeling more applicable to drug development and clinical therapy.

## Figures and Tables

**Table 1:** Summary of common disease progression models in oncology. Representative curves are also included to illustrate the changes in tumor size over time.

Model	Mathematical Equation	Representative Curve	References
Exponential	$\frac{dY}{dt}=\lambda_{0}\times Y\left(t\right)$ Y(t)—the tumor mass at time t λ_0_—the exponential rate of growth	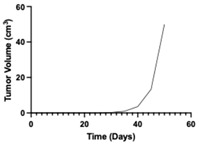	[62,63,64,65,66,67]
Gompertz	$\frac{dY}{dt}=Y\left(t\right)\;$× (α−β ×$\;ln(\frac{Y\left(t\right)}{Y_{0}}$)) Y_0_—the tumor mass at time zero Y(t)—the tumor mass at time t α—the growth constant β—the natural loss constant	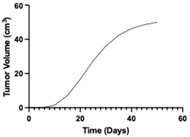	[19,63,65,68,69]
Logistic	$\frac{dY}{dt}=\alpha \;$×$\;Y\left(t\right)-\beta \;$×${\;Y\left(t\right)}^{2}$ Y(t)—the tumor mass at time t α—the growth constant β—the natural loss constant	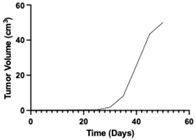	[63,64,65,70,71]
Bertalanffy	$\frac{dY}{dt}=\alpha \;$×${Y\left(t\right)}^{\frac{2}{3}}-\beta \;$×Y (t) Y(t)—the tumor mass at time t α—the growth constant β—the natural loss constant	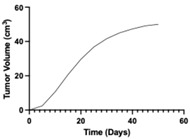	[63,64,65,72,73,74]
Simeoni	$\frac{dY}{dt}=\frac{\lambda_{0}\times Y\left(t\right)}{[1+(\frac{\lambda_{0}}{\lambda_{1}}\;\times {\;Y(t))}^{\psi }{]}^{\;\frac{1}{\psi }}}$ Y(t)—the tumor mass at time t λ_0_—the exponential rate of tumor growth λ_1_—the linear rate of tumor growth ψ—the shape parameter controls the nonlinearity and smoothness of the tumor growth function	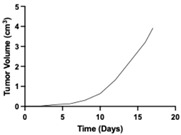	[20]
Yamazaki	$\frac{dY}{dt}=K_{in}\;\times \left(1-\frac{Y\left(t\right)}{{TG}_{50}+Y\left(t\right)}\right)\times \;Y\left(t\right)-K_{out}\times \;Y\left(t\right)$ Y(t)—the tumor mass at time t K_in_—the first-order tumor growth rate constant TG_50_—the tumor mass that inhibits 50% of the growth rate K_out_—the first-order tumor loss rate constant	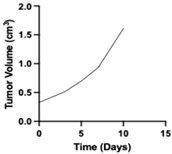	[75]
Koch	$\frac{dY}{dt}=\frac{2\;\times \lambda_{0}\;\times \lambda_{1}\;\times Y\left(t\right)}{\lambda_{1}+2\;\times \lambda_{0}\;\times Y\left(t\right)}$ Y(t)—the tumor mass at time t λ_0_—the exponential rate of tumor growth λ_1_—the linear rate of tumor growth	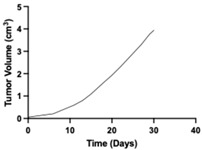	[34]

**Table 3:** Common PK-PD models for combination therapy in oncology.

Model	Combination Factors	Equation	References
Koch	Interaction factor ψ	$\frac{dx_{1}}{dt}=\frac{2\cdot \lambda_{0}\times \lambda_{1}\times {x_{1}(t)}^{2}}{\left(\lambda_{1}+2\times \lambda_{0}\times x_{1}\left(t\right)\right)\times w}-(k_{2A}\;{\times \;C}_{A}\left(t\right)+k_{2B}\;{\times \;C}_{B}\left(t\right)\cdot \psi )\cdot x_{1}(t)$	[34]
		$\frac{dx_{2}}{dt}=\left(k_{2A}\;{\times \;C}_{A}\left(t\right)+k_{2B}\;{\times \;C}_{B}\left(t\right)\times \psi \right)\times x_{1}(t)-k_{1}^{\prime }\times x_{2}(t)$	
		$\frac{dx_{3}}{dt}=k_{1}^{\prime }\times x_{2}(t)-k_{1}^{\prime }\times x_{3}(t)$	
		$\frac{dx_{4}}{dt}=k_{1}^{\prime }\times x_{3}(t)-k_{1}^{\prime }\times x_{4}(t)$	
		$w\left(t\right)=x_{1}\left(t\right)+x_{2}\left(t\right)+x_{3}\left(t\right)+x_{4}(t)$	
		$x_{1}\left(0\right)=w(0);\;x_{2}\left(0\right)=x_{3}\left(0\right)=x_{4}\left(0\right)=0$ $k_{1}^{\prime }$—the transient rate constant after combination treatment $k_{2A},\;k_{2B}$—the potency of the drug A or B λ_0_—the exponential rate of tumor growth λ_1_—the linear rate of tumor growth x_1_—the mass of proliferating cells x_2_, x_3_, x_4_ —the mass of non-proliferating cells at different damage states $w\left(t\right)$—the tumor mass at time t $C\left(t\right)$—the plasma concentration of drug at time t	
Terranova	Interaction term $\nu_{ij}$ Interaction parameter γ	$\frac{dx_{00}}{dt}=\frac{\lambda_{0}\times x_{00}(t)}{[1+(\frac{\lambda_{0}}{\lambda_{1}}\;{{\times \;w}_{0}(t))}^{\psi }{]}^{\;\frac{1}{\psi }}}-\left(k_{2A}\times C_{A}\left(t\right)+k_{2B}\times C_{B}\left(t\right)+\nu_{11}\right)\;{\times \;x}_{00}$	[35]
		$\frac{dx_{ij}}{dt}=u_{Aij}+u_{Bij}+\nu_{ij},\;i+j>0$	
		$W(t)=\sum_{i=0}^{3}\sum_{j=0}^{3}x_{ij}\left(t\right)$	
		$u_{Aij}=\left\{\begin{array}{c} \;0,\;i=0\\ k_{2A}\;{\times \;C}_{A}\left(t\right)\times x_{i-1,j}-k_{1A}x_{ij},\;i=1\\ \;k_{1A}x_{i-1,j}-k_{1A}x_{ij},\;i=2,\;3 \end{array}\right.$	
		$u_{Bij}=\left\{\begin{array}{c} \;0,\;j=0\\ k_{2B}\;{\times \;C}_{B}\left(t\right)\;\times \;x_{i,j-1}-k_{1B}x_{ij},\;j=1\\ \;k_{1B}\;\times \;x_{i,j-1}-k_{1B}x_{ij},\;j=2,\;3 \end{array}\right.$	
		$\nu_{ij}=\left\{\begin{array}{c} \gamma \;{\times \;C}_{A}\left(t\right)\;{\times \;C}_{B}\left(t\right)\;{\times \;x}_{00},\;i=j=1\\ \;0,\;otherwise \end{array}\right.$ $k_{1A},k_{1B}$—the transient rate constant after drug A or B treatment $k_{2A},k_{2B}$—the potency of the drug A or B λ_0_—the exponential rate of tumor growth λ_1_—the linear rate of tumor growth $x_{00}$—the mass of the proliferating cells $x_{ij}$—the mass of non-proliferating cells at different damage states $u_{Aij},$ $u_{Bij}$—the drug effect of A or B at stage ij γ—the interaction parameter $w\left(t\right)$—the tumor mass at time t $C\left(t\right)$—the plasma concentration of drug at time t	
Choi-Yu	Contribution (or combination) factors α,β	$\frac{dx_{1}}{dt}=\frac{2\times \lambda_{0}\times \lambda_{1}\times {x_{1}(t)}^{2}}{\left(\lambda_{1}+2\times \lambda_{0}\times x_{1}\left(t\right)\right)\times w}-\left(\alpha \times k_{2A}{\times C}_{A}\left(t\right)+\beta \times k_{2B}{\times C}_{B}\left(t\right)\right)\times x_{1}(t)$	[33]
		$\frac{dx_{2}}{dt}=\left({\alpha \;\times \;k}_{2A}\;{\times \;C}_{A}\left(t\right)+{\beta \;\times \;k}_{2B}\;{\times \;C}_{B}\left(t\right)\right)\;{\times \;x}_{1}(t)-k_{1}^{\prime }\;\times \;x_{2}(t)$	
		(All other equations remain the same as Koch’s)	

## Data Availability

The datasets generated during the current study are available from the corresponding author on reasonable request.

## Abbreviations

The following abbreviations are used in this manuscript:

AUC	area under the curve
CAR-T	Chimeric Antigen Receptor T cell therapy
CDM	cell distribution model
CL	clearance
C_max_	the maximum drug concentration
cMet	c-mesenchymal–epithelial transition factor
CI	combination index
EC50	half-maximal effective concentration
EGFR	Epidermal Growth Factor Receptor
FDA	Food and Drug Administration
FLT3	Feline McDonough Sarcoma-like tyrosine kinase 3
Gli1	glioma-associated oncogene 1
HER2	human epidermal growth factor receptor 2
IDR	indirect response
IC50	half-maximal inhibitory concentration
ka	absorption rate constant
ke	elimination rate constant
k_g_	a tumor growth function
K_in_	the first-order tumor growth rate constant
K_out_	the first-order tumor loss rate constant
mAb	monoclonal antibody
MIDD	model-informed drug development
NCA	non-compartmental analysis
NSCLC	non-small cell lung cancer
PBPK	physiologically based pharmacokinetic
PD	pharmacodynamics
PD-L1	programmed death-ligand 1
PK	pharmacokinetics
QSP	quantitative systems pharmacology
SDM	signal distribution model
Smo	Smoothened
t_1/2_	half-life
TG_50_	the tumor size that inhibits 50% of the growth rate
TGI	tumor growth inhibition
TKI,	tyrosine kinase inhibitor
T_max_	time to maximum concentration
TSC	tumor-static concentration
TV	tumor volume
V_d_	Volume of Distribution
